# Beyond desalinization: root interactions with halophyte *Suaeda salsa* reshape soybean rhizosphere metabolite-microbiome networks

**DOI:** 10.3389/fpls.2026.1847720

**Published:** 2026-07-21

**Authors:** Shiqi Wang, Jinbiao Liu, Jiliang Zheng, Mingfang Hu, Changyan Tian

**Affiliations:** 1 State Key Laboratory of Desert and Oasis Ecology, Xinjiang Institute of Ecology and Geography, Chinese Academy of Sciences (CAS), Urumqi, China; 2 University of Chinese Academy of Sciences, Beijing, China; 3 Agricultural College, Heilongjiang Bayi Agricultural University, Daqing, China; 4 Xinjiang Xinlianxin Energy Chemical Co., Ltd., Changji, China

**Keywords:** halophyte, intercrop, phytoremediation, salinity, untargeted metabolomics

## Abstract

**Introduction:**

Halophyte-based intercropping may involve root interactions beyond desalinization in alleviating salt stress in glycophytes.

**Methods:**

To elucidate the mechanisms of root interactions enhancing soybean (*Glycine max*) salt tolerance intercropping with *Suaeda salsa*, we analyzed rhizosphere metabolomes and bacterial communities under two salt treatments (no additional NaCl, S1; 3 g kg^–1^ NaCl, S3) and three root interaction modes: (1) plastic barrier (no root interactions), (2) nylon mesh barrier (root interactions only), and (3) no barrier (root interactions with potential salt redistribution).

**Results:**

Both NL and NS significantly increased soybean biomass compared with PL under both salt treatments, with no significant difference between NL and NS. Under S3, NL increased soybean biomass by 80% relative to PL without significantly changing soil electrical conductivity, accompanied by increases in rhizosphere carbohydrates, organic acids, betaine, flavonoids, and putative plant growth-promoting bacteria (PGPB). Although soybean rhizosphere Na^+^ decreased under NS compared with PL and NL, this was accompanied by reduced putative PGPB abundance and no further biomass increase compared with NL. Coumestrol, trehalose-6-phosphate, and isopentenyl pyrophosphate (IPP) were identified as hub metabolites associated with soybean rhizosphere microbial community structure, with the IPP-related module representing a potential component of the salt-response network. Intercropping also increased available phosphorus (AP) in both species’ rhizospheres, with increases in soybean associated with organic acids and those in *S. salsa* associated with rhizosphere pH shifts and putative PGPB changes.

**Discussion:**

These findings indicate that root interactions enrich salt−tolerance−related metabolites in the soybean rhizosphere and suggest potential metabolic-microbial coupling underlying intercropping−induced salt tolerance.

## 1 Introduction

Soil salinization poses a significant threat to the sustainability of agricultural production, affecting over 1 billion hectares worldwide across more than 100 countries, emerging as a critical challenge to global food security (Rozema and Flowers, 2008; Hopmans et al., 2021). Under the dual pressures of climate change-induced extreme weather events and intensified land use due to population growth, saline-affected areas have been expanding at an alarming rate of approximately 1–2 million hectares per year (Ivushkin et al., 2019; Eswar et al., 2021; Hopmans et al., 2021). Against this backdrop, halophytes—a unique group of plants naturally adapted to high-salinity environments—have garnered increasing attention. These plants not only provide multiple economic benefits (e.g., food, fodder, fiber, and medicinal uses) but exhibit the potential to effectively desalinize soil through salt accumulation in their tissues (Rozema and Flowers, 2008; Litalien and Zeeb, 2020; Wang et al., 2021). Given these advantages, incorporating halophytes into intercropping systems provides a dual-functional strategy that achieves both economic sustainability and salt stress mitigation for co-cultivated glycophytes (Ben Hamed et al., 2021).

The productivity of intercropping systems is determined not only by interspecific competition and complementarity in resource utilization, but also profoundly regulated by root interactions (Duchene et al., 2017). Even in the absence of direct root contact, neighboring plants can influence each other through underground signaling networks, enhancing nutrient availability (e.g., N, P, and Fe) (Zuo et al., 2000; Li et al., 2003a, 2003b; Li et al., 2004; Chen et al., 2007; Shao et al., 2021; Hei et al., 2022) and suppressing soil-borne diseases (Lv et al., 2018; Zhou et al., 2023; Lv and Yan, 2024). Traditionally, the beneficial effects of halophyte-based intercropping on salt stress mitigation have been mainly attributed to physical desalinization via halophyte-mediated salt uptake (Simpson et al., 2018; Albaho and Green, 2020; Ben Hamed et al., 2021). However, recent evidence suggests that improvements in glycophyte salt tolerance can also occur without significant changes in soil salinity (Jurado-Mañogil et al., 2023; Jurado et al., 2024; Wang et al., 2025a). This suggests that root-mediated biological processes constitute an additional, yet insufficiently understood, pathway contributing to plant salt tolerance.

These belowground interactions are increasingly recognized to be mediated by rhizosphere metabolites, which are low-molecular-weight compounds derived from plant roots, associated microbes, and soil organic matter, and represent an integrated direction of plant-microbe-environment interactions (Wen et al., 2022; Shi et al., 2023; Baker et al., 2024; Jiang et al., 2024). Representative rhizosphere metabolites included organic acids (e.g., citric and malic acids), which mobilize poorly soluble nutrients such as phosphate (Shen et al., 2011; Yang et al., 2012; Goswami et al., 2014). Compatible solutes (e.g., proline, trehalose, and glycine betaine) helped maintain osmotic balance and stabilize cellular structures under salinity stress (Iordachescu and Imai, 2008; Suárez et al., 2008; Hu et al., 2020; Sarkar and Sadhukhan, 2022; Li et al., 2025). Flavonoids mitigated oxidative stress through reactive oxygen species scavenging (Chapman et al., 2019). Importantly, these metabolites exert dual functions by directly regulating plant physiological responses and simultaneously shaping microbial community assembly (Shi et al., 2023; Wen et al., 2023; Baker et al., 2024; Jiang et al., 2024). This dual role establishes rhizosphere metabolites as central mediators of plant stress adaptation (Wen et al., 2022, 2023; Baker et al., 2024; Jiang et al., 2024).

Soybean (*Glycine max*) is a globally important staple and cash crop, providing approximately 70% of the world’s plant protein and 29% of vegetable oil (http://www.soystats.com). Salt stress represents one of the major abiotic factors limiting its yield and quality (Phang et al., 2008; Han et al., 2023). *Suaeda salsa*, a widely distributed euhalophyte, demonstrates remarkable soil desalinization and functional improvement through aboveground biomass harvesting and soil structure modification (Wang et al., 2021; Li et al., 2023; Wang et al., 2025b). Its root exudates can reshape rhizosphere microbial communities and enrich salt-tolerant taxa, thereby influencing soil ecological functions (Peng, 2021; Wang et al., 2025b). Moreover, microbial communities associated with *S. salsa* have been shown to promote growth and salt tolerance in non-host plants, suggesting strong interspecific facilitation potential (Yuan et al., 2016; Wang et al., 2022a). Recent work further demonstrates that intercropping soybean with *S. salsa* enhances biomass and antioxidant capacity even in the absence of significant soil desalinization (Wang et al., 2025a), suggesting the involvement of belowground mechanisms. Here, based on integrated metabolome-microbiome analyses and root separation experiments, this study addresses three key questions: (i) How do root interactions reshape soybean rhizosphere metabolic profiles and microbial community structures? (ii) How do metabolites regulate microbial communities? (iii) Which metabolite-microbe interaction patterns are associated with soybean salt tolerance? Moving beyond the conventional focus on physical desalinization, this study helps elucidate the biological basis of intercropping-induced salt tolerance, suggesting that plant-microbe interactions may represent an additional regulatory mechanism complementing desalinization effects. This work also identifies potential targets for optimizing saline intercropping systems and developing microbiome-based strategies to enhance crop performance under salt stress.

## 2 Materials and methods

### 2.1 Experimental design and sample collection

The pot experiment was conducted from June to August 2024 at Xinjiang Xinlianxin Energy Chemical Co., Ltd.’s artificial intelligence greenhouse (25°C ± 2°C day/20°C ± 2°C night). Plastic pots (25 × 15 × 23 cm) filled with 7.2 kg of soil collected from the surface layer (0–20 cm) of agricultural fields at Fukang Station (44.3°N, 87.9°E), Chinese Academy of Sciences, Xinjiang Uygur Autonomous Region. The soil fundamental physicochemical properties were analyzed as follows: pH 8.4, salinity 1.4 g kg^–1^, electrical conductivity (EC) 0.32 dS m^–1^, organic matter content 13.8 g kg^–1^, available phosphorus (AP) 45.6 mg kg^–1^, NH_4_^+^-N 10.4 mg kg^–1^, NO_3_^–^N 79.0 mg kg^–1^, and field capacity 25%. Basal fertilizers were applied at rates of 0.2 g kg^–1^ N and 0.1 g kg^–1^ P.

The experiment included two salt treatments: no extra salt addition (S1) and soil salt content of 3 g kg^−1^ (S3, added as NaCl), along with three root interaction modes (Li et al., 2003a): (i) complete separation with plastic barrier (PL); (ii) 400-mesh (37 μm) nylon net barrier (NL) allowing exchange of root exudates and microorganisms while restricting direct root contact and continuous soil mass flow; and (iii) no separation (NS) permitting unrestricted root interactions and allowing potential changes in rhizosphere soil properties, including spatial redistribution of soil salts, as well as exchange of root exudates and microorganisms.

*S. salsa* was first sown on one side of the pot, followed by soybean sowing on the opposite side after 30 days. At the V1 stage of soybean growth, seedlings were thinned to maintain 4 soybean plants and 6 *S. salsa* plants per pot.

Deionized water was replenished every two days using the weighing method to maintain soil moisture at 75% of field capacity. Due to significant differences in water consumption between the two species, pots of PL were completely separated and weighed individually for precise soil moisture control. Although the nylon mesh prevented continuous mass flow caused by water competition, salt redistribution could still occur through irrigation. To minimize salt transfer from soybean zones to *S. salsa* in NL, two preventive measures were implemented: (i) partial watering of the *S. salsa* zone before complete irrigation, and (ii) pre-sampling measurement of soil EC values in soybean zones, with only pots of NL exhibiting EC values comparable to PL being selected for subsequent analysis. Each treatment ultimately comprised 25 pots, with five pots pooled into one biological replicate, resulting in five biological replicates per treatment.

Plant and soil samples were collected 30 days after soybean emergence. After harvesting the aboveground plant parts, they were oven-dried at 105°C for 30 minutes, then dried at 65°C for 48 hours before weighing to determine aboveground biomass. Rhizosphere soil was collected from within 2 mm of the root surface, with one portion stored at –80°C for analysis of rhizosphere metabolites and bacterial communities, and another portion air-dried for analysis of soil physicochemical properties.

### 2.2 Soil physicochemical properties

The soil solution was extracted at a 1:5 soil-to-water ratio, with pH determined using a calibrated pH meter (FiveEasy Plus FE28, Mettler-Toledo, Switzerland), EC measured using a conductivity meter (H1 2315, HANNA, Italy), soluble Na^+^ and K^+^ concentrations determined by atomic absorption spectroscopy (iCE 3000 series, Thermo Fisher Scientific, USA), and Ca^2+^ and Mg^2+^ measured via chemical titration. NO_3_^–^N was extracted with 0.01 M calcium chloride and analyzed using an auto-analyzer (AA3, Bran Luebbe, Hamburg, Germany), while AP (Olsen-P) was extracted with 0.5 M NaHCO_3_ and quantified by the molybdenum-antimony colorimetric method (Cary 60, Agilent Technologies, USA).

### 2.3 Analysis of rhizosphere soil metabolome

Rhizosphere soil samples were collected for untargeted metabolomics analysis. Sample preparation and data analysis were performed following standard protocols provided by Wuhan Metware Biotechnology Co., Ltd. The experiment employed ultra-performance liquid chromatography (Thermo Vanquish, Thermo Fisher Scientific, USA) coupled with tandem mass spectrometry (Thermo Q Exactive HF-X, Thermo Fisher Scientific, USA) for LC-MS/MS analysis and metabolite quantification.

All samples were analyzed using two LC/MS methods with detection in both electrospray ionization positive (ESI+) and negative (ESI–) modes. Chromatographic separation was achieved using a Waters ACQUITY Premier HSS T3 column (1.8 μm, 2.1 × 100 mm) with mobile phases consisting of 0.1% formic acid in water (A) and 0.1% formic acid in acetonitrile (B), employing the following gradient elution program: 0–2 min (5%→20% B), 2–5 min (20%→60% B), 5–6 min (60%→99% B), 6–7.5 min (hold at 99% B), 7.5–7.6 min (99%→5% B), and 7.6–10 min (equilibration at 5% B). The chromatographic conditions included a column temperature of 40°C, flow rate of 0.4 mL min^–1^, and injection volume of 4 μL, with identical gradient programs for both ionization modes.

Mass spectrometric detection utilized full-scan MS and data-dependent MS^n^ scan modes, covering a mass range of m/z 75–1000 at a resolution of 35, 000. The electrospray voltage was set at +3.5 kV for ESI+ and –3.2 kV for ESI–, with sheath gas and auxiliary gas flows of 30 Arb and 5 Arb, respectively. The ion transfer tube and vaporizer temperatures were maintained at 320°C and 300°C. Data-dependent acquisition employed dynamic exclusion (3 s), stepped collision energies (30/40/50 V), intensity threshold of 1 × 10^6^ cps, and Top N (N = 10) priority acquisition strategy.

Raw data were converted to mzXML format using ProteoWizard software (v3.0.24), followed by peak picking, alignment, and retention time correction using XCMS. Peaks with missing rates >50% in any group were removed, and peak areas were normalized using Support Vector Regression. Metabolite annotation was performed based on retention time (RT), precursor ions (Q1), and MS/MS fragmentation patterns by matching against self-built and public databases (HMDB, https://hmdb.ca; KEGG, https://www.kegg.jp). The data presented in figures represent merged results from both ionization modes.

### 2.4 DNA extraction, amplicon sequencing, and bioinformatic analyses

Genomic DNA was extracted from 0.5 g of fresh soil using the Omega M5635–02 Mag-Bind Soil DNA Kit (Omega Bio-Tek, USA), strictly following the manufacturer’s protocol. DNA concentration and purity were assessed via a NanoDrop 2000 spectrophotometer (Thermo Fisher Scientific, USA). The V3–V4 region of bacterial 16S rDNA was amplified using forward primer 338F (5’-ACTCCTACGGGAGGCAGCA-3’) and reverse primer 806R (5’-GGACTACHVGGGTWTCTAAT-3’), with 7 bp sample-specific barcodes for multiplex sequencing. PCR reactions contained 5 μL 5× buffer, 0.25 μL FastPfu DNA polymerase (5 U μL^–1^), 2 μL dNTPs (2.5 mM), 1 μL each forward/reverse primer (10 μM), 1 μL DNA template, and 14.75 μL ddH2O. Thermal cycling conditions: 98°C for 5 min; 25 cycles of 98°C for 30 s, 53°C for 30 s, 72°C for 45 s; and 72°C for 5 min final extension.

Paired-end sequencing was performed on the Illumina NovaSeq platform (USA) at Shanghai Personal Biotechnology Co., Ltd. Raw sequences were processed in QIIME2 2019.4 using the DADA2 plugin for quality control, denoising, merging, and chimera removal as described by Callahan et al. (2016). Taxonomic annotation of amplicon sequence variants (ASVs) employed the classify-sklearn naive Bayes classifier against the SILVA Release 132 database. Microbial richness was calculated based on the ASVs table using “qiime diversity alpha-rarefaction” within QIIME2.

Following the approach described by Peng et al. (2025), putative plant growth-promoting bacteria (PGPB) with functions including nitrogen fixation, phosphate solubilization, indole-3-acetic acid (IAA) production, 1-aminocyclopropane-1-carboxylate deaminase (ACCD) activity, and siderophore secretion were analyzed using a local 16S rRNA gene database (Chao et al., 2016). Local BLAST+ (v2.3.0) alignments were conducted with an E-value cutoff of 1 × 10^–10^, retaining matches with ≥ 97% similarity. Only the top-hit reference sequence was retained for downstream analysis (Zhalnina et al., 2018; Peng et al., 2025).

To validate the reliability of the PGPB 16S rRNA database and BLAST-based comparison, the nitrogen-fixing gene nifH was quantified by quantitative PCR (qPCR) as described by Chao et al. (2016), using primers nifHF (5’-AAAGGYGGWATCGGYAARTTCACCAC-3’) and nifHRb (5’-TGSGCYTTGTCYTCRCGGATBGGCAT-3’). Each 25 μL qPCR reaction contained 12.5 μL Maxima SYBR Green qPCR Master Mix (Thermo Fisher Scientific, USA), 1 μM of each primer, and 20 ng of DNA template, with the volume adjusted to 25 μL using RT-PCR grade water. The thermal profile included an initial denaturation at 95 °C for 15 min; 40 cycles of 94 °C for 1 min, annealing for 1 min (starting at 65 °C and decreasing by 1 °C per cycle to 50 °C), and extension at 72 °C for 1 min; followed by a final extension at 72 °C for 10 min. All reactions were performed in triplicate. The purified PCR products were cloned into the pMD18-T vector (TaKaRa, Japan) to generate the standard curve for qPCR. The nifH gene copy numbers were normalized to the DNA mass (ng) and compared with the relative abundance of nitrogen-fixing bacteria obtained from BLAST alignments. The Spearman correlation coefficients between the two independent methods were high and statistically significant (**Supplementary Figure S1**; *R*^2^ = 0.72, *p* < 0.01), providing partial validation for the effectiveness of the PGPB 16S rRNA database and BLAST-based identification used in this study.

### 2.5 Statistical analysis

All data are presented as mean ± standard error based on five biological replicates. Two-way ANOVA was used to analyze the interaction between salinity and the root interaction modes on biomass and soil physicochemical properties. Differences among root interaction modes under the same salt treatment were assessed using one-way ANOVA followed by Duncan’s *post hoc* test (*p* < 0.05).

Orthogonal partial least squares-discriminant analysis (OPLS-DA) was performed to maximize group separation in the metabolomics data. Model validity was assessed using 200 permutation tests, and Q^2^, R^2^Y, and their corresponding permutation p-values are reported for each comparison. Differential metabolites (DEMs) were identified based on variable importance in projection (VIP > 1) and fold change (|log2FC| > 2). Raw p-values obtained from independent-sample *t*-tests were adjusted for multiple comparisons using the Benjamini-Hochberg method, and metabolites with a false discovery rate (FDR) < 0.1 were considered statistically significant. This threshold was applied to balance statistical stringency and the detection of biologically relevant metabolites in untargeted metabolomic datasets.

Procrustes analysis and Mantel tests evaluated associations between metabolites and bacterial communities. MetagenomeSeq was applied to identify significantly different ASVs in soybean rhizospheres under S3 (NL vs. PL and NS vs. PL), then a correlation network of S3 integrated differential ASVs, DEMs, and soil properties using Spearman’s rank correlation (*p* < 0.05). The correlation threshold (|r| > 0.84) was determined using Random Matrix Theory (RMT) implemented in the” RMThreshold” R package, and intra-group correlations were excluded from the network. Network modules were defined as topological communities detected using the fast greedy modularity optimization algorithm implemented in the “igraph” R package (Baker et al., 2024). Putative keystone metabolites and ASVs were identified based on within-module (zi) and among-module (pi) connectivity. Nodes were classified as module hubs (zi > 2.5), connectors (pi > 0.62), or network hubs (both criteria met). Finally, linear regression of z-transformed variables was performed to quantify relationships between soil AP and chemical properties, metabolites, and phosphate-solubilizing PGPB.

All analyses were performed in R (v4.3.0) using packages (vegan, metagenomeSeq, igraph, ggClusterNet), with visualizations generated in R and Cytoscape (v3.10.0).

## 3 Results

### 3.1 Biomass and soil properties

**Figure 1:**
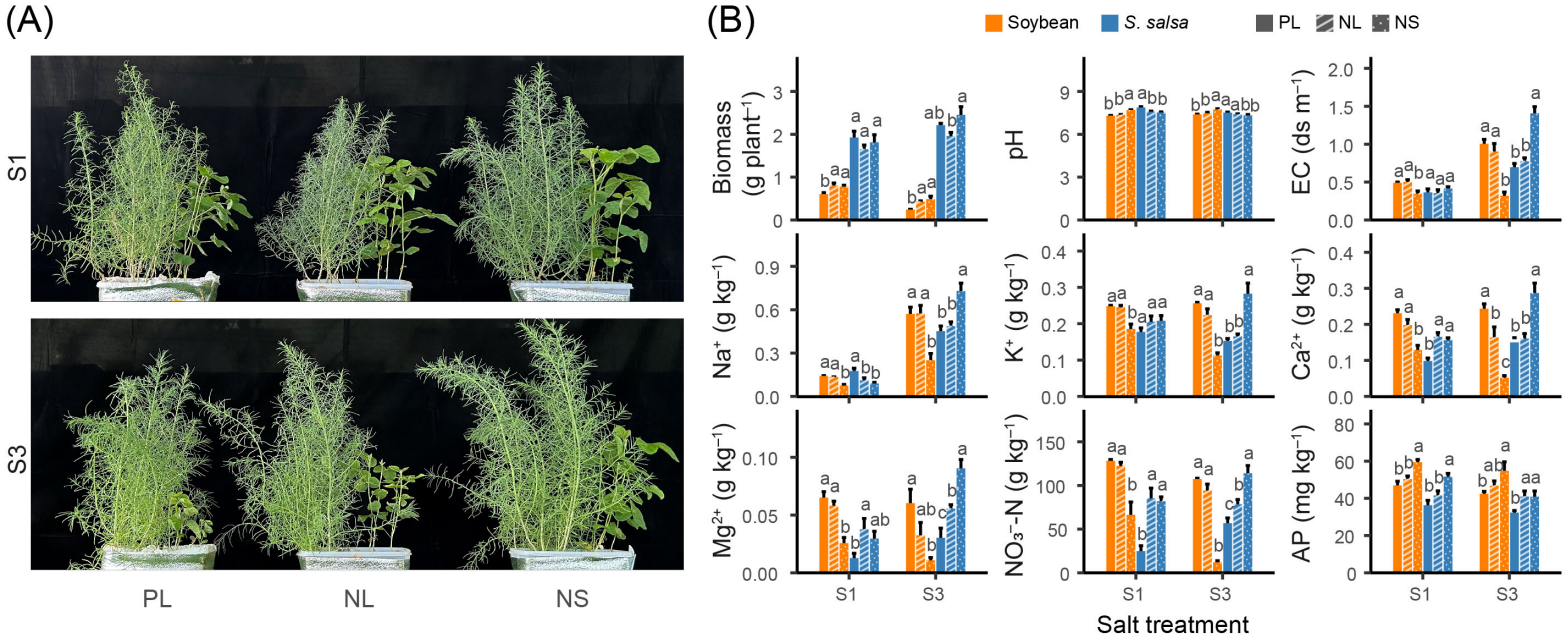
Plant growth and soil chemical properties. **(A)** Schematic diagram of plant growth; **(B)** Biomass and soil chemical properties. PL, plastic barrier separation; NL, nylon mesh separation; NS, no separation. Different lowercase letters in **(B)** indicate significant differences (p = 0.05) among root interaction modes for the same species under the same salt treatment.

Salt stress significantly inhibited soybean growth, with soybean biomass under S3 treatment being markedly lower than under S1 treatment, while *S. salsa* showed the opposite trend (*p* < 0.05; **Figure 1A**; **Table 1**). Under both salt treatments, soybean biomass in NL and NS treatments was significantly higher than in PL, with increases of approximately 30% under S1 and 80–100% under S3 (*p* < 0.05), while no significant difference was observed between NS and NL (*p* > 0.05; **Figure 1B**). For *S. salsa*, biomass showed no significant differences among the three intercropping modes under S1, whereas under S3, NL was significantly lower than NS, though neither differed significantly from PL (**Figure 1B**).

**Table 1:** Effects of salinity, root interactions, and their interaction effects on biomass and soil chemical properties.

	Soybean			*Suaeda salsa*		
	S	RI	S × RI	S	RI	S × RI
Biomass	**	**	ns	**	ns	ns
pH	*	**	ns	**	**	ns
EC	**	**	**	**	**	**
Na^+^	**	**	**	**	**	**
K^+^	**	**	**	ns	**	**
Ca^2+^	*	**	*	**	**	**
Mg^2+^	*	**	ns	**	**	**
NO_3_^−^-N	**	**	ns	**	**	*
AP	ns	**	ns	*	**	ns

S, Salt treatment; RI, Root interaction mode. *, **, and ns denote significance at *p* = 0.05, *p* = 0.01, and non-significance, respectively.

No significant interaction between salt treatment and root interaction mode on rhizosphere soil pH of the two species (*p* > 0.05; **Figure 1B**; **Table 1**). In the soybean rhizosphere, pH exhibited an increasing trend from PL to NL to NS, with NS being significantly higher than PL and NL (*p* < 0.05; **Figure 1B**). Conversely, *S. salsa* rhizosphere pH followed the opposite pattern, with PL being higher than NL and NS (*p* < 0.05; **Figure 1B**).

Under both salt treatments, soybean rhizosphere EC in NS was significantly lower than in PL and NL, decreasing by approximately 30% under S1 and 67% under S3 (*p* < 0.05; **Figure 1B**), with no significant difference between PL and NL (*p* > 0.05). For *S. salsa*, rhizosphere EC showed no significant variation among root interaction modes under S1, whereas under S3, NS was significantly higher than PL and NL by ~91% (*p* < 0.05; **Figure 1B**), with no difference between PL and NL (*p* > 0.05). Changes in soil Na^+^, K^+^, Ca^2+^, Mg^2+^, and NO_3_^–^N generally mirrored EC trends, except that soybean rhizosphere Na^+^ under S3 was significantly higher than S1 in NS, while K^+^, Ca^2+^, Mg^2+^, and NO_3_^–^N were significantly lower under S3 than S1 (**Figure 1B**).

No significant interaction between salt treatment and root interaction mode on rhizosphere soil AP of the two species (*p* > 0.05; **Figure 1B**, **Table 1**). Under both salt treatments, the rhizosphere AP content of both plant species exhibited a gradual increasing trend across PL, NL, and NS treatments, with NS showing significantly higher AP levels than PL.

### 3.2 Rhizosphere differential metabolites

**Figure 2:**
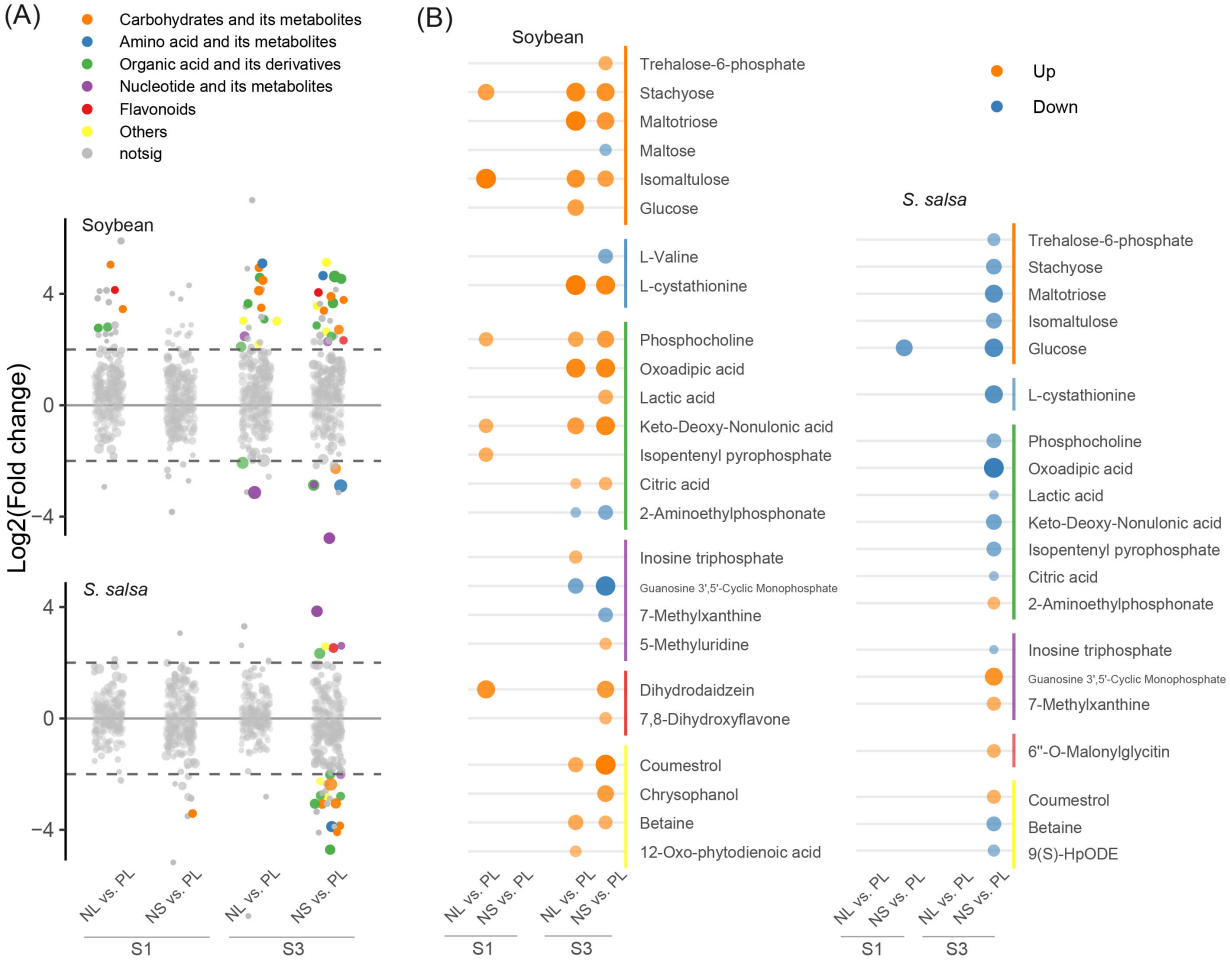
Rhizosphere differential metabolites of different comparisons. **(A)** Volcano plot of metabolites; **(B)** Differential metabolite profiles. Point size in **(A)** represents –log_2_*(p*-value); in **(B)** represents |log_2_(fold change)|. PL, plastic barrier separation; NL, nylon mesh separation; NS, no separation.

**Figure 3:**
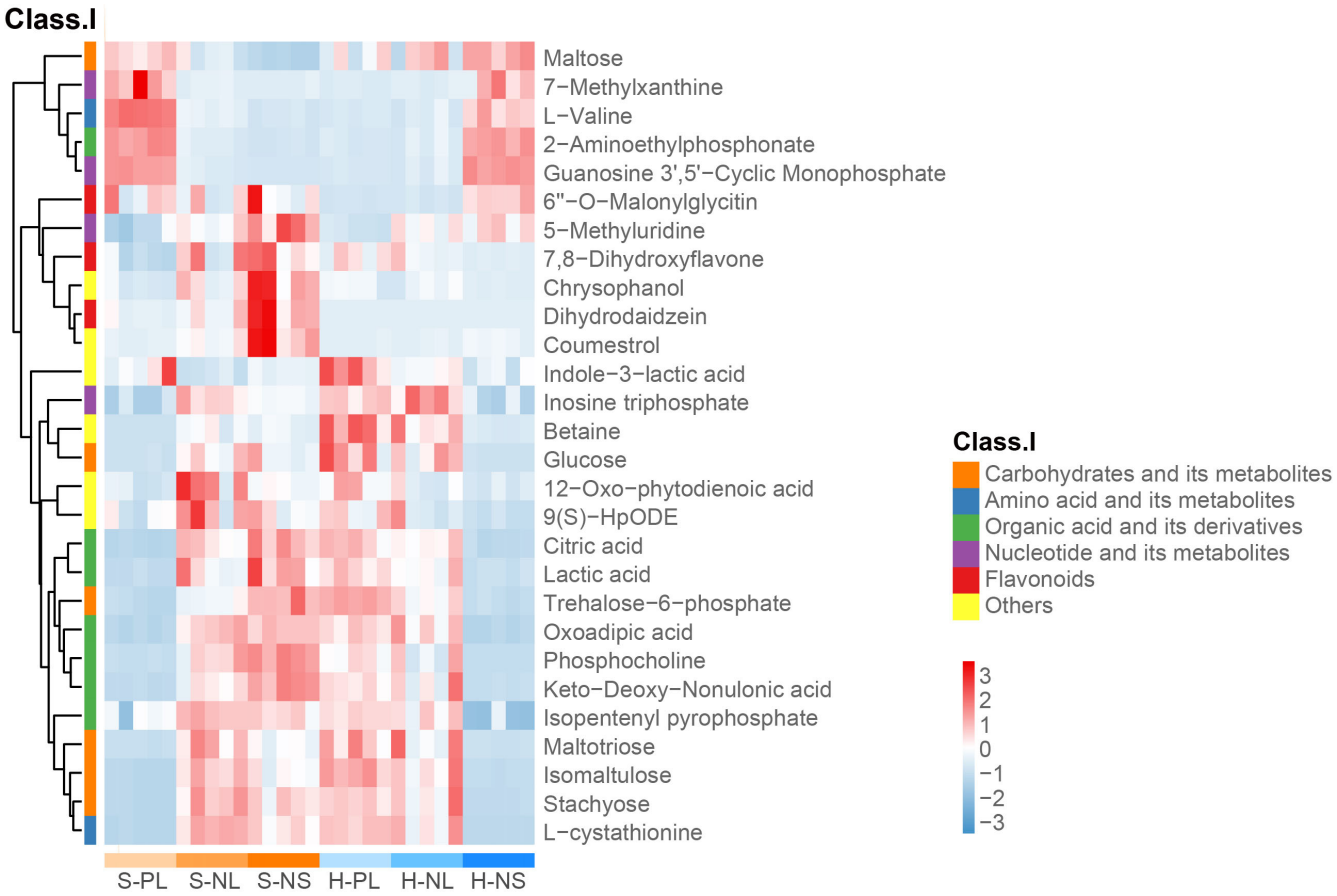
Clustered heatmap of differential metabolites under S3 salt treatment. S, Soybean; H, *Suaeda salsa*. PL, Plastic barrier separation; NL, Nylon mesh separation; NS, No separation.

OPLS-DA models for the four group comparisons showed good descriptive and predictive ability, with R^2^Y(cum) ranging from 0.989 to 0.998 and Q^2^(cum) ranging from 0.719 to 0.893. Two hundred Y-permutation tests demonstrated that the original models exhibited higher R^2^Y and Q^2^ values than those obtained from permuted models. The Q^2^ intercepts ranged from –0.346 to –0.301, and the R^2^Y intercepts ranged from 0.647 to 0.668. These results indicate that the models were not overfitted and support their validity for further interpretation. A total of 371 metabolites were identified. Under S1 treatment, compared with PL, NL showed 6 upregulated and no downregulated metabolites, whereas NS showed no differentially abundant metabolites (0 up, 0 down). Under S3, NL exhibited 13 upregulated and 2 downregulated metabolites relative to PL, while NS displayed 16 upregulated and 5 downregulated metabolites (**Figure 2**). Except for flavonoids, these upregulated metabolites were more abundant in *S. salsa* rhizosphere than soybean in PL under S3 treatment, with NL and NS displaying similar patterns under S3 (**Figure 3**).

The upregulated metabolites primarily included carbohydrates and derivatives (isomaltulose, maltotriose, stachyose, trehalose-6-phosphate, glucose), organic acids and derivatives (isopentenyl pyrophosphate, IPP, phosphocholine, citric acid, oxoadipic acid, keto-deoxy-nonulonic acid, lactic acid), flavonoids (dihydrodaidzein, 7, 8-dihydroxyflavone), and others (betaine, chrysophanol, 12-oxo-phytodienoic acid, coumestrol) (**Figures 2**, **3**). In contrast to soybean, *S. salsa* rhizosphere showed downregulation trends for all these metabolites in NL and NS versus PL except coumestrol (**Figures 2**, **3**).

### 3.3 Rhizosphere bacterial communities

**Figure 4:**
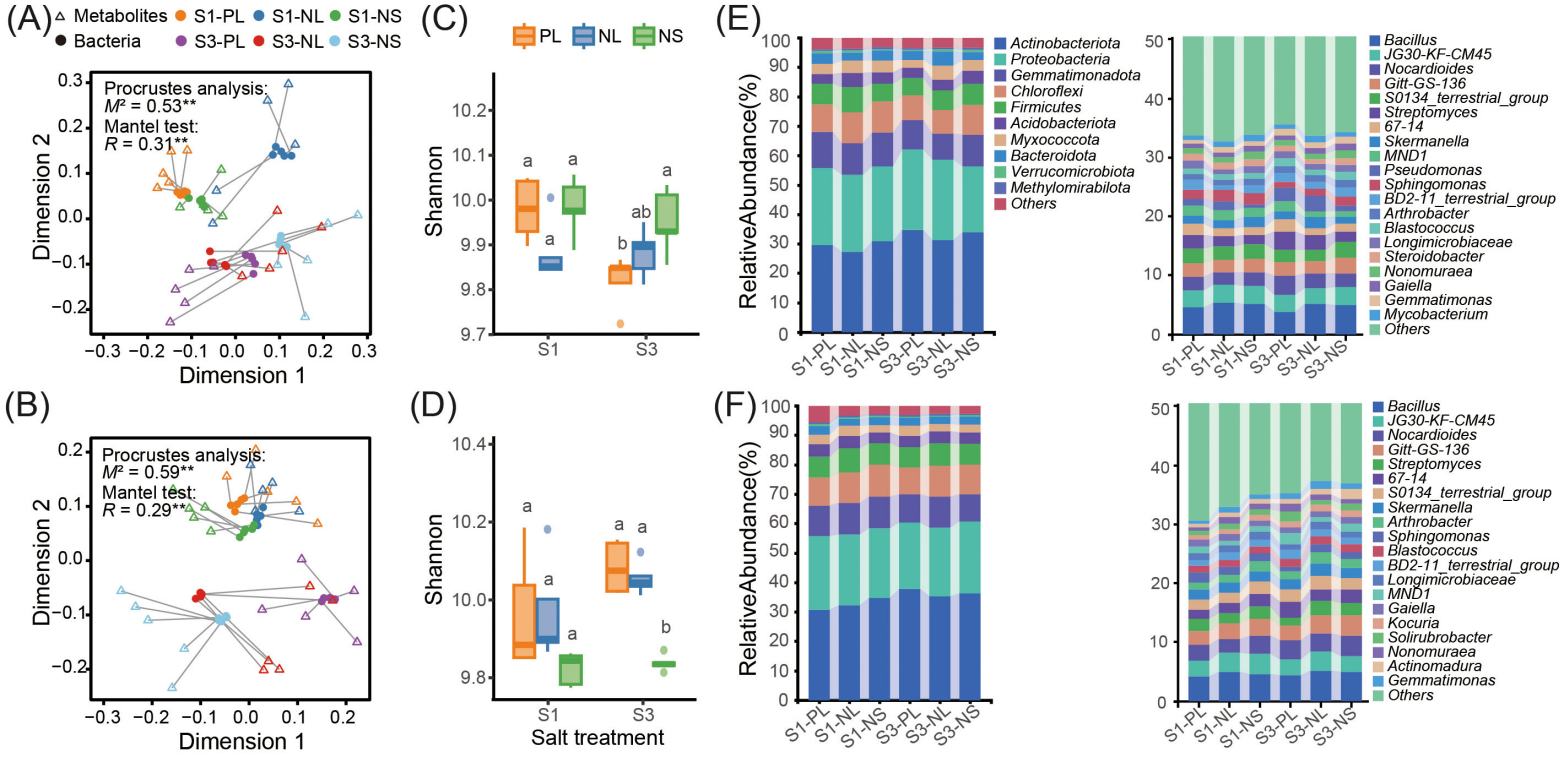
Rhizosphere bacterial communities. **(A, B)** Procrustes analysis and Mantel test assessing metabolite-microbiome correlations; **(C, D)** Bacterial community diversity indices; **(E, F)** Relative abundances of the top 10 bacterial phyla and top 20 genera. Panels **(A, C, E)** Soybean; Panels **(B, D, F)** *Suaeda salsa*. PL, Plastic barrier separation; NL, Nylon mesh separation; NS, No separation. Different lowercase letters in **(C, D)** indicate significant differences (*p* = 0.05) among root interaction modes under the same salt treatment.

Significant rhizosphere metabolome-microbiome correlations occurred in soybean (Procrustes *M*^2^ = 0.53, *p* < 0.01; Mantel *R* = 0.31, *p* < 0.01; **Figure 4A**) and *S. salsa* (Procrustes *M*^2^ = 0.59, *p* < 0.01; Mantel *R* = 0.29, *p* < 0.01; **Figure 4B**). For soybean, PL rhizosphere bacterial diversity (Shannon) under S3 treatment was significantly lower than under S1 treatment. Within S3 treatment, diversity showed an increasing trend from PL to NL to NS, with NS reaching significant levels (*p* < 0.05; **Figure 4C**). Under S3 treatment, compared to PL, NL decreased *Actinobacteria* and *Chloroflexi* while increasing *Bacteroidota*, *Firmicutes*, *Myxococcota*, and *Verrucomicrobiota*; NS reduced *Proteobacteria* and *Bacteroidota* but increased *Gemmatimonadota*, *Chloroflexi*, *Myxococcota*, and *Verrucomicrobiota* (**Figure 4E**). *S. salsa* rhizosphere exhibited opposite diversity trends to soybean, with NS under S3 being significantly lower than PL and NL, while PL and NL showed no significant difference (**Figure 4D**). Compared to PL, both NL and NS decreased *Actinobacteria* and *Myxococcota* but increased *Proteobacteria*, *Chloroflexi*, and *Firmicutes* (**Figure 4F**). At the genus level, *Bacillus*, *Sphingomonas*, *Gaiella*, and *Nonomuraea* exhibited relatively higher abundance under both NL and NS treatments than under PL across all salinity levels. In contrast, *Nocardioides*, *Streptomyces*, 67-14, and BD2-11_terrestrial_group showed relatively lower abundance under intercropping treatments regardless of salinity level. *Skermanella* was more abundant under NL than under PL under S3 treatment, whereas under NS the opposite trend was observed.

**Figure 5:**
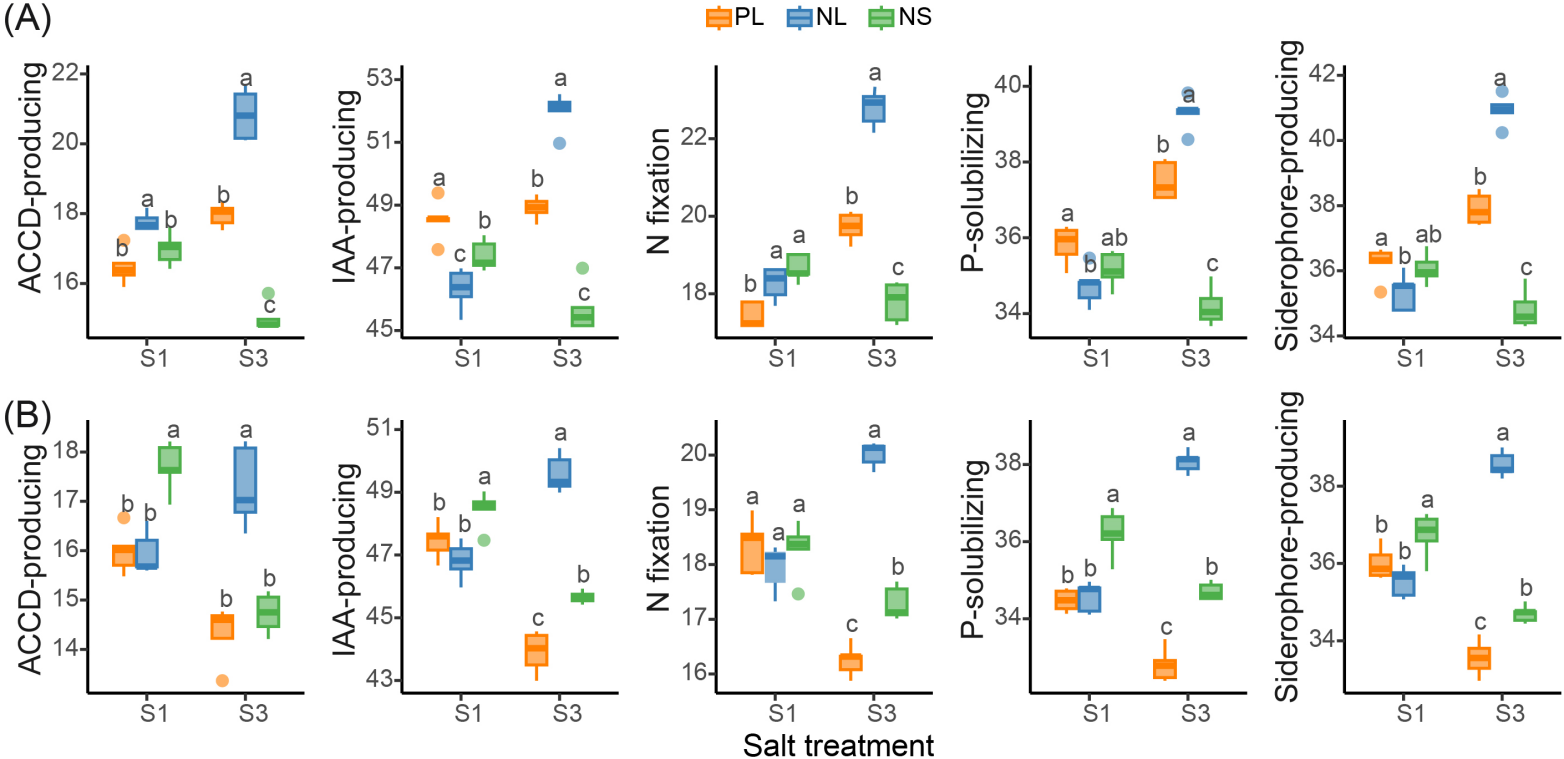
Relative abundance of potential rhizosphere plant growth-promoting bacteria. **(A)** Soybean; **(B)** *Suaeda salsa*. ACCD, 1-aminocyclopropane-1-carboxylate deaminase; IAA, Indole-3-acetic acid; PL, plastic barrier separation; NL, nylon mesh separation; NS, no separation. Different lowercase letters indicate significant differences (*p* = 0.05) among root interaction modes under the same salt treatment.

Under S1 treatment, soybean rhizosphere showed significantly higher relative abundance of potential PGPB with ACC deaminase activity in NL compared to PL and NS, with no difference between PL and NS (**Figure 5A**). IAA-producing PGPB were highest in PL, followed by NS and NL. Nitrogen-fixing PGPB were significantly lower in PL than NL and NS, while phosphate-solubilizing and siderophore-producing PGPB were higher in PL than NL (**Figure 5A**). Under S3 treatment, all PGPB functional groups (ACC deaminase production, IAA synthesis, nitrogen fixation, phosphate solubilization, and siderophore production) followed the same pattern, where relative abundances were significantly higher in NL than PL (*p* < 0.05), with both exceeding NS (*p* < 0.05). For *S. salsa* under S1 treatment, except for nitrogen fixation, PGPB abundances were significantly higher in NS than in NL and PL (*p* < 0.05), with no significant difference observed between NL and PL (*p* > 0.05; **Figure 5B**). Under S3 treatment, ACC deaminase-producing PGPB exhibited significantly higher relative abundance in NL than in PL and NS (*p* < 0.05), with no PL-NS difference detected. For other functional groups, abundance hierarchies followed NL > NS > PL, with all inter-group differences being statistically significant (*p* < 0.05; **Figure 5B**).

### 3.4 Association analysis between DEMs and bacterial communities

**Figure 6:**
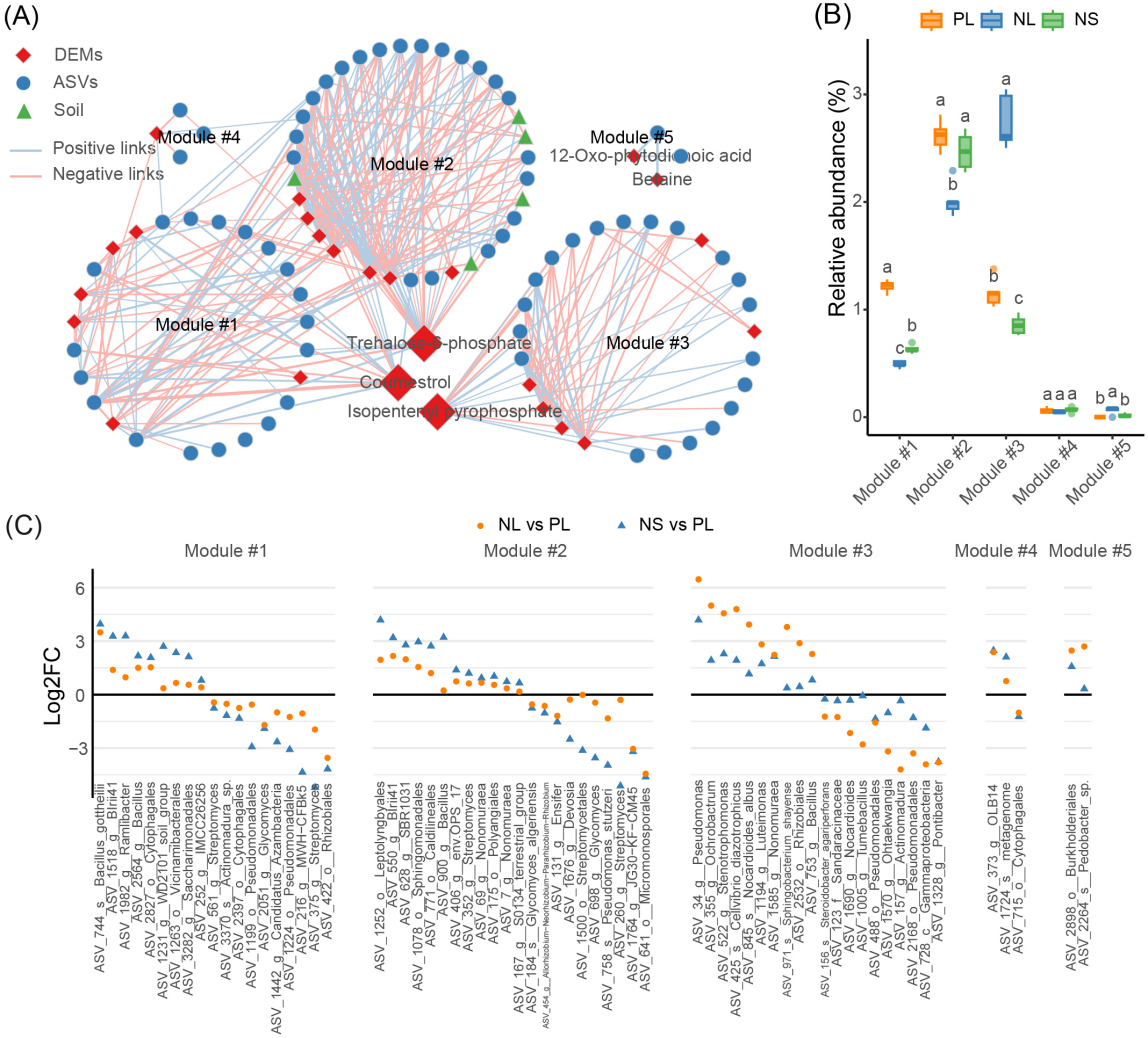
Co-occurrence network of differential metabolites and ASVs in soybean rhizosphere under S3 salt treatment. **(A)** Co-occurrence network of differential metabolites and ASVs; **(B)** Relative abundance of module-associated bacteria; **(C)** Fold changes of module bacteria across comparison groups. Highlighted nodes in **(A)** represent network hubs or module hubs. Different lowercase letters in **(B)** indicate significant differences (*p* = 0.05) among root interaction patterns. PL, Plastic barrier separation; NL, Nylon mesh separation; NS, No separation.

Under S3 treatment, soybean rhizosphere soil properties, differential metabolites, and ASVs with significant changes in NL and NS compared to PL were used to construct a correlation network, revealing five modules (**Figure 6A**). Module 1 was characterized by the key node coumestrol, along with other metabolites including genistein, daidzin, 7, 8-dihydroxyflavone, sophoricoside, dihydrodaidzein, chrysophanol, 5-methyluridine, lactic acid , and citric acid. The bacterial relative abundance in this module was significantly higher in PL than in NL and NS (*p* < 0.05; **Figure 6B**), with upregulated ASVs in NL and NS primarily belonging to *Bacillus* (ASV 744, ASV 2564; **Figure 6C**).

Module 2 featured trehalose-6-phosphate (T6P) as the module hub, accompanied by L-valine, 2-aminoethylphosphonate, phosphocholine, keto-deoxy-nonulonic acid, guanosine 3’, 5’-cyclic monophosphate, maltose, and oxoadipic acid (**Figure 6A**). The bacterial community in this module exhibited significantly higher abundance in PL than in NS, and both PL and NS were significantly higher than NL (*p* < 0.05; **Figure 6B**).

Module 3 was driven by IPP, forming predominantly positive correlations with other metabolites, including inosine triphosphate (ITP), isomaltulose, maltotriose, L-cystathionine, stachyose, and glucose (**Figure 6A**). The bacterial relative abundance in this module was significantly higher in NL than in PL, and both NL and PL were higher than NS (*p* < 0.05; **Figure 6B**).

Module 4 contained the metabolite 7-methylxanthine, while Module 5 included 12-oxo-phytodienoic acid (12-OPDA) and betaine (**Figure 6A**), with bacterial relative abundance significantly higher in NL than in PL and NS, and no significant difference between PL and NS (**Figure 6B**). Upregulated ASVs in Module 5 were assigned to *Burkholderiales* (ASV 2898) and *Pedobacter* (ASV 2264) (**Figure 6C**).

### 3.5 Correlations between soil available phosphorus and soil pH, metabolites, and PGPB

**Figure 7:**
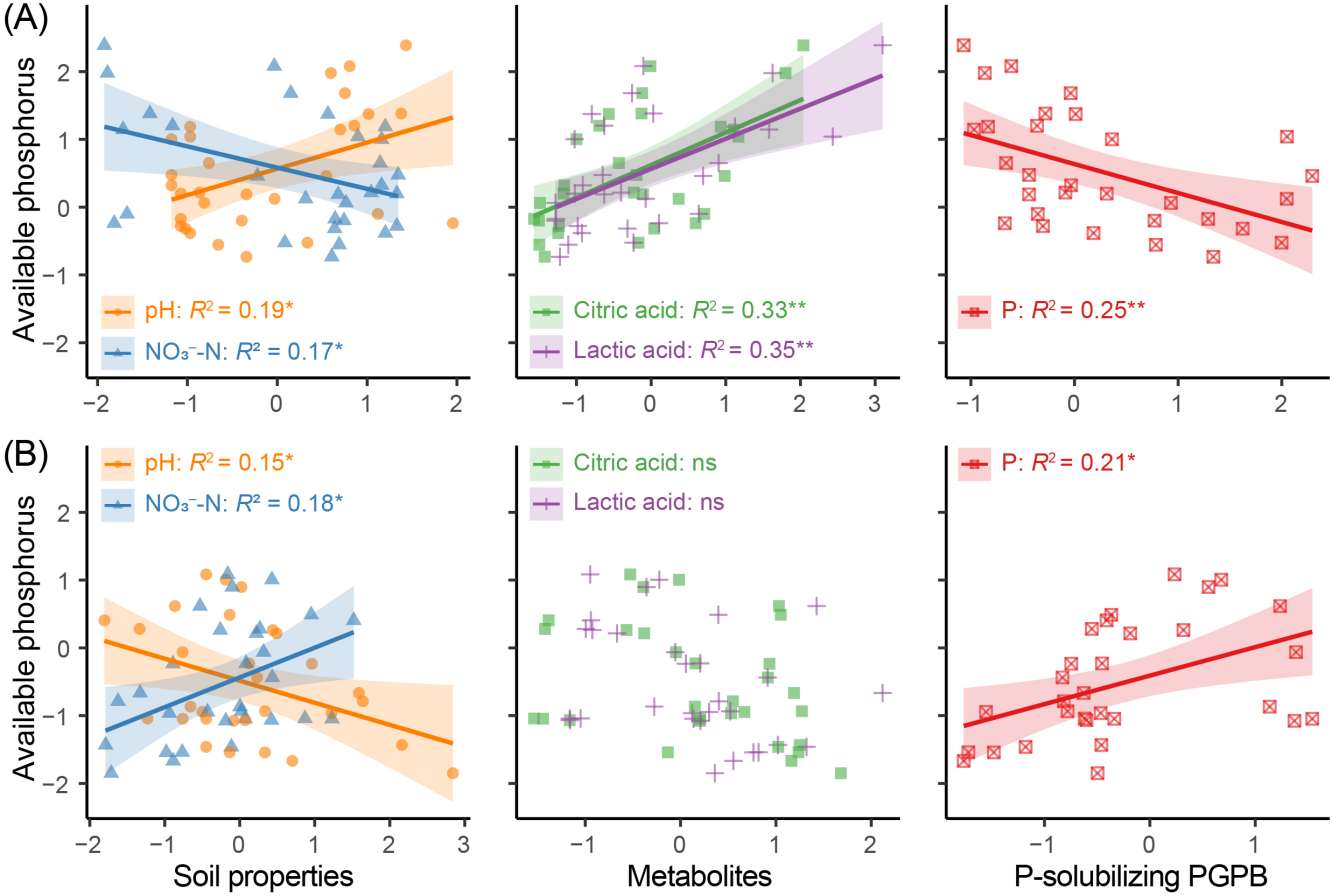
Linear relationships between soil available phosphorus and chemical properties, metabolites, and phosphate-solubilizing PGPB. **(A)** Soybean; **(B)** *Suaeda salsa*. Data were z-score normalized. *, **, and ns denote significance at *p* = 0.05, *p* = 0.01, and non-significance, respectively.

In the soybean rhizosphere, AP exhibited a significant positive correlation with the abundance of citric acid (*R*^2^ = 0.33, *p* < 0.01) and lactic acid (*R*^2^ = 0.35, *p* < 0.01), while showing negative correlations with phosphate-solubilizing PGPB abundance (*R*^2^ = 0.25, *p* < 0.01; **Figure 7**) and positively correlated with soil pH (*R*^2^ = 0.19, *p* < 0.05). In contrast, *S. salsa* rhizosphere displayed an opposite pattern: AP showed positive correlations with phosphate-solubilizing PGPB (*R*^2^ = 0.21, *p* < 0.05) abundance and NO_3_^−^-N (*R*^2^ = 0.18, *p* < 0.05), and a negative correlation with pH (*R*^2^ = 0.15, *p* < 0.05). However, no significant linear relationship was observed between AP and citric acid or lactic acid in *S. salsa* (*R*^2^ = 0.15, *p* > 0.05; **Figure 7**).

## 4 Discussion

### 4.1 Complementary utilization of soil salinity

Interspecific complementarity refers to differences in resource utilization between intercropped species in terms of temporal, spatial, and chemical properties, which is one of the key mechanisms underlying intercropping advantages (Duchene et al., 2017). In this study, soybean growth was inhibited by salinity, whereas *S. salsa* was promoted under saline conditions (**Figure 1**). The significant reduction in soil salinity in the soybean rhizosphere and the concurrent increase in *S. salsa* rhizosphere after intercropping demonstrated their interspecific complementarity in salinity utilization, thereby facilitating species coexistence and yield improvement (**Figure 1**). However, it should be noted that while *S. salsa* absorbs salt, it simultaneously competes for water and mobile nutrients. Such trade−offs are consistent with previous observations in halophyte−based systems (Simpson et al., 2018; Wang et al., 2022b, 2022c). Thus, appropriate field water and fertilizer management is required to mitigate interspecific competition.

### 4.2 Enrichment of rhizosphere salt-tolerance metabolites

Root interactions showed no significant effects on soybean rhizosphere soil properties (**Figure 1**) or plant salt content (Wang et al., 2025a), but significantly increased biomass and antioxidant capacity (**Figure 1**; Wang et al., 2025a), suggesting that root interactions enhance soybean salt tolerance under saline conditions without directly modifying bulk soil salinity. In intercropping systems, plants can alter secondary metabolite secretion in response to neighboring plants (Zhou et al., 2023), thereby regulating rhizosphere nutrient cycling and recruiting plant growth-promoting bacteria (Shi et al., 2023; Zhou et al., 2023; Baker et al., 2024; Jiang et al., 2024). In this study, soybean rhizosphere metabolic profiles were structured by root interactions with *S. salsa* in salinity conditions, with a general increase in stress-related metabolites (**Figures 2**, **3**). Key compounds—including trehalose-6-phosphate (T6P), betaine, stachyose, flavonoids, coumestrol, chrysophanol, and 12-oxo-phytodienoic acid (12-OPDA)—have been associated with multiple stress-related processes, such as osmotic regulation, redox homeostasis, and signaling modulation (Iordachescu and Imai, 2008; Jeon et al., 2012; Sarkar and Sadhukhan, 2022; Song et al., 2024; Deng et al., 2025). Collectively, these metabolite shifts suggest a more pronounced stress-defense strategy in soybean. In contrast, *S. salsa* exhibited an opposite metabolic trend in intercropping, with reduced levels of trehalose-6-phosphate, betaine, flavonoids, and organic acids, suggesting a shift from osmotic defense toward biomass expansion. This pattern may be related to the greater salt availability in intercropping. As a euhalophyte, *S. salsa* performs optimally under high salinity (200 mM NaCl), where abundant inorganic ions support osmotic adjustment, whereas low-salinity conditions may restrict the ion availability required for efficient osmotic regulation (Song et al., 2009; Liu et al., 2026). Similar to root interaction-mediated facilitation under abiotic stress in intercropping systems (Brooker et al., 2015), soybean and *S. salsa* exhibited different adaptive strategies under shared saline conditions. Soybean tended to adopt a more pronounced stress-defense strategy, whereas *S. salsa* may act as an environmental modulator, potentially alleviating rhizosphere salinity. This division of labor likely contributes to improved soybean performance and system-level stability under intercropping conditions.

### 4.3 Microbial community alterations

Multiple relationships exist between the enrichment of putative potential plant growth-promoting bacteria (PGPB) (ACCD production, IAA synthesis, siderophores, phosphorus solubilization, nitrogen fixation) and plant salt tolerance, with core mechanisms spanning plant physiological regulation and soil nutrient activation (Goswami et al., 2014; Chao et al., 2016; Sarkar et al., 2018; Choudhury et al., 2021; Haroon et al., 2022; Li et al., 2025). In this study, root interactions enhanced soybean rhizosphere microbial diversity and significantly increased the abundance of predicted PGPB (**Figures 4**, **5**), suggesting that belowground microbial shifts may contribute to improved salt tolerance and nutrient acquisition in intercropped soybean. Previous studies have demonstrated that rhizosphere microbial communities are jointly shaped by soil physicochemical properties and root exudates across diverse plant systems (Yang et al., 2012; Zhalnina et al., 2018; Baker et al., 2024; Wang et al., 2025b). In the present study, the absence of significant differences in soybean rhizosphere salinity and pH between NL and PL treatments suggests that the putative PGPB enrichment was more likely associated with intercropping−induced changes in soybean root exudates (**Figures 1**, **2**). Similar to earlier findings that greater exudate complexity enhances microbial recruitment and network complexity (Yang et al., 2012; Shi et al., 2023; Zhou et al., 2023; Jiang et al., 2024; Wang et al., 2024a). Furthermore, plant-plant proximity has been reported to facilitate microbial exchange via root exudates or microbial migration (Vora et al., 2021; Zhou et al., 2023; Wang et al., 2024a). In this context, *S. salsa* may be associated with shifts in the relative abundance of putative PGPB in the soybean rhizosphere.

Although desalinization increased bacterial diversity, it was associated with a reduced relative abundance of putative PGPB in the soybean rhizosphere. The salt-tolerant traits of PGPB require substantial metabolic investment, such as the synthesis of compatible solutes for maintaining cellular osmotic balance under salt stress (Detkova and Boltyanskaya, 2007). When salt stress is alleviated, these adaptive traits may become less selectively advantageous, potentially reducing their relative competitiveness within the microbial community. PGPB are known to contribute to plant growth promotion through multiple mechanisms, including nutrient mobilization, phytohormone regulation, and stress alleviation (Sarkar et al., 2018; Choudhury et al., 2021; Li et al., 2025). In this context, the reduced abundance of putative PGPB may be associated with the absence of additional soybean biomass gains under NS compared with NL (**Figure 1**). However, the actual expression of these PGPB requires further validation via isolate-based functional assays, as 16S-based predictions do not guarantee phenotypic expression at the strain level.

Intercropping with *S. salsa* reshaped the soybean rhizosphere bacterial community, with community assembly influenced by both salinity gradients and belowground plant-plant interactions. At the phylum level, consistent enrichment of Firmicutes under NL and NS treatments, together with increases in Myxococcota and Verrucomicrobiota, indicates that intercropping promotes biotically associated restructuring of the rhizosphere in addition to salinity-driven environmental filtering. At the genus level, *Bacillus*, *Sphingomonas*, *Gaiella*, and *Nonomuraea* showed relatively higher abundance under both NL and NS treatments across salinity levels, indicating that these taxa may be more closely associated with intercropping-driven root interactions than with salinity variation. Functionally, *Bacillus* is widely recognized for its plant growth-promoting and stress-tolerance traits, including osmotic regulation and antioxidant enhancement (Upadhyay et al., 2011; Goswami et al., 2014; Haroon et al., 2022; Han et al., 2023). The other three genera—*Sphingomonas*, *Gaiella*, and *Nonomuraea*—have been implicated in carbon utilization and organic matter turnover (Haichar et al., 2008; Lipun et al., 2019; Amon et al., 2023), collectively suggesting a potential role in soybean rhizosphere carbon cycling. In contrast, *Nocardioides*, *Streptomyces*, 67-14, and BD2-11_terrestrial_group showed relatively lower abundance under intercropping treatments regardless of salinity level, suggesting greater sensitivity to altered root-derived resources and interspecific microbial competition. Collectively, these results suggest that soybean-*S. salsa* intercropping drives a reorganization of the rhizosphere microbiome. This process leads to the enrichment of interaction-favored taxa, while reducing the relative abundance of competition-sensitive groups.

### 4.4 Rhizosphere metabolite-related microbial assembly

Rhizosphere metabolites shape bacterial community assembly (Zhalnina et al., 2018; Zhou et al., 2023; Baker et al., 2024). Correlation network analysis enables the identification of system−level salt−tolerance modules and helps highlight putative hub metabolites with potential regulatory roles (Baker et al., 2024; Wang et al., 2025b). Here, “modules” refer to groups of metabolites and amplicon sequence variants (ASVs) that co-varied across treatments and were hypothesized to participate in coordinated responses to salinity and root interactions, although their exact biochemical interactions remain to be experimentally validated. The relative microbial abundance in Module 1 was associated with root interactions regardless of desalinization effects (**Figure 6**). The metabolite-microbe interaction network revealed co-occurrence patterns between coumestrol-centered metabolites along with other upregulated compounds, including flavonoids, which showed strong associations with the enrichment of *Bacillus* (ASV 744, ASV 2564). Coumestrol and flavonoids have been reported to inhibit pathogens (Lozovaya et al., 2004; Jeon et al., 2012; Zhou et al., 2023) and have been repeatedly detected in association with biocontrol-associated microbes such as *Bacillus* (Goswami et al., 2014; Wen et al., 2023; Zhou et al., 2023; Wang et al., 2024b). These results suggest that Module 1 may reflect a biocontrol-related metabolite-microbe co-occurrence pattern.

The relative microbial abundance in Module 2 was significantly lower in NL than NS, with changes in T6P-centered metabolites being associated with the enrichment of osmotolerant microbes such as *Leptolyngbyales* (ASV 1252) that synthesize extracellular polysaccharides. As a core osmotic regulator and carbon signaling metabolite, T6P plays an important role in coordinating plant-microbe osmotic adjustment under salt stress (Detkova and Boltyanskaya, 2007; Suárez et al., 2008; Iturriaga et al., 2009; Sarkar and Sadhukhan, 2022; Wang et al., 2024b). Stress-induced accumulation of trehalose-related metabolites has been reported to be associated with bacterial community composition (Lian et al., 2021; Liang et al., 2021; Xu et al., 2026), while exogenous trehalose application has been shown to promote beneficial microbial groups (Xu et al., 2025, 2026). In a dose−dependent manner, T6P-centered metabolic shifts likely act as selective filters that shape the enrichment of adapted microbial taxa, rather than directly determining microbial abundance (Lian et al., 2021; Xu et al., 2026). Concurrently, the upregulation of phosphocholine may contribute to membrane stability by serving as a precursor in phospholipid biosynthesis and participating in membrane lipid remodeling. It has also been implicated in ABA-related signaling pathways associated with osmotic stress responses (He et al., 2022). Given that NS faces stronger water competition than NL, these results suggest that Module 2 may reflect a metabolite-microbe association related to osmotic adjustment and shaped by selective filtering of T6P-centered metabolites.

Module 3 showed higher relative microbial abundance under NL than under PL, but lower abundance under NS (**Figure 6**). Its abundance was positively correlated with carotenoid content and negatively correlated with H_2_O_2_ levels in soybean leaves (**Supplementary Figure 2**). Network analysis further identified isopentenyl pyrophosphate (IPP) as a hub metabolite within this module, which co-occurred with inosine triphosphate (ITP). IPP is known as a terpenoid precursor for carotenoid synthesis (Chapman et al., 2019), while ITP has been implicated in DNA damage responses and salicylic acid-mediated signaling networks (Straube et al., 2022). These patterns suggest Module 3 may be associated with soybean antioxidant-related adjustments. Although Module 5 involved fewer microbial taxa, it positively correlated with soybean salt tolerance (**Supplementary Figure 2**). 12-OPDA, a precursor of jasmonic acid biosynthesis, may contribute to ROS detoxification via glutathione-associated antioxidant pathways (Ribot et al., 2008; Zhu et al., 2023), while betaine alleviates ion toxicity by stabilizing protein structures and osmotic balance (Hu et al., 2020; Li et al., 2025). Module 5 metabolites (12-OPDA and betaine) were significantly upregulated in NL, accompanied by enrichment of beneficial bacteria *Pedobacter* sp. and *Burkholderiales*. These changes correlated with increased glutathione peroxidase and glutathione reductase activities and decreased malondialdehyde content in soybean (**Supplementary Figure 2**; Wang et al., 2025a), suggesting that this module may be associated with improved redox homeostasis under intercropping conditions. Notably, *Pseudomonas* ASV 34 (Module 3), *Pedobacter* sp. ASV 2264, and *Burkholderiales* ASV 2898 (Module 5) represent potential PGPB candidates that are valuable for further isolation and validation.

### 4.5 Soil phosphorus activation

In alkaline soils, phosphorus availability is regulated by soil pH, root exudates, and microbial activity through coupled geochemical and biological processes (Shen et al., 2011; Goswami et al., 2014; Li et al., 2020; Yang et al., 2022). These processes are also influenced by nitrogen inputs, which may indirectly affect AP through soil pH regulation (Li et al., 2020). In this study, intercropping significantly increased AP in both soybean and *S. salsa* rhizospheres, but the underlying associated processes appeared to differ between the two species. In the soybean rhizosphere, the increase in AP was associated with elevated levels of citric acid and lactic acid. These compounds are well known to enhance phosphorus solubilization through chelation of cations (e.g., Ca^2+^ and Fe^3+^) and dissolution of insoluble phosphate minerals (Ström et al., 2005; Shen et al., 2011). By contrast, the increase in AP observed in the *S. salsa* rhizosphere coincided with shifts in rhizosphere pH and changes in the relative abundance of putative phosphate-solubilizing PGPB. These patterns are consistent with previous findings that co−occurring species often employ distinct phosphorus−acquisition strategies (Ceulemans et al., 2017; Phoenix et al., 2020). Complementary phosphorus utilization between intercropped species has been widely recognized as a key driver of intercropping advantages (Li et al., 2003a, 2003b; Li et al., 2004, 2016). Our results suggest that intercropping may promote phosphorus availability through species−specific, complementary mechanisms, which warrant further validation with direct measurements of P transformation processes.

## 5 Conclusion

Both desalinization and root interactions alleviated soybean salt stress in saline soils when intercropped with *S. salsa*. Root interactions significantly increased soybean salt tolerance, elevated salt-tolerance-related metabolites in the rhizosphere (including trehalose-6-phosphate, betaine, organic acids, and flavonoids), and boosted the relative abundance of putative PGPB, as inferred from 16S rRNA-based taxonomy. Desalinization created a low-salinity environment for soybean, but it reduced the relative abundance of putative PGPB in the rhizosphere. Coumestrol, trehalose-6-phosphate, and isopentenyl pyrophosphate (IPP) emerged as core rhizosphere metabolites associated with the assembly of distinct metabolite-microbial modules in soybean rhizosphere. Among these, the IPP-associated module showed a stronger correlation with soybean salt tolerance; experimental validation is still required to confirm the roles of the associated microbial taxa. Additionally, intercropping significantly enhanced AP in both species’ rhizospheres through potentially distinct mechanisms: organic acid secretion in soybean versus putative PGPB enrichment and pH reduction in *S. salsa*. Nonetheless, phosphorus transformation processes and predicted PGPB functions require confirmation through direct measurements of phosphorus dynamics and isolate-based functional assays. Finally, complementary patterns of salt utilization and competition for mobile nutrients between the two species offer practical insights for field deployment of this intercropping system.

## Data Availability

The datasets presented in this study can be found in online repositories. The names of the repository/repositories and accession number(s) can be found below: https://www.ncbi.nlm.nih.gov/, PRJNA1449004.
