# Supplementary material for: Beyond desalinization: root interactions with halophyte Suaeda salsa reshape soybean rhizosphere metabolite-microbiome networks
Source: Front Plant Sci. 2026 Jul 21;17:1847720. doi: 10.3389/fpls.2026.1847720 (PMC13433301; doi:10.3389/fpls.2026.1847720)
Supplement: Supplementary file 1 [file SupplementaryFile1.docx]

Supplementary Material

# 1 Supplementary Figures

**Supplementary Figure 1.** Comparison of *nifH* gene quantification using 16S rRNA gene analysis (via PGPB database and BLAST) and qPCR. (A) *nifH* gene copy numbers under different treatments; (B) Correlation of the two independent methods for *nifH*.

**Supplementary Figure 2.** Correlation between the relative abundance of module-associated bacteria and soybean leaf physiological indices. Chlt: total chlorophyll; Car: carotenoids; MDA: malondialdehyde; SOD: superoxide dismutase; POD: guaiacol peroxidase; CAT: catalase; APX: ascorbate peroxidase; GPX: glutathione peroxidase; GR: glutathione reductase.
